# Prognostic value of pretreatment neutrophil-to-lymphocyte ratio independent of comorbidity burden in melanoma

**DOI:** 10.1016/j.jdin.2026.02.012

**Published:** 2026-03-16

**Authors:** Kevin Chun-Kai Chiu, Yang Lee, Ming-Hsien Lin, Yi-Hua Liao, Jau-Yu Liau, Chia-Yu Chu, Yi-Shuan Sheen

**Affiliations:** a Department of Dermatology, National Taiwan University Hospital, Taipei, Taiwan; b Department of Medical Education, National Taiwan University Hospital, Taipei, Taiwan; c Department of Surgery, National Taiwan University Hospital Hsin-Chu Branch, Hsin-chu, Taiwan; d Department of Dermatology, National Taiwan University College of Medicine, Taipei, Taiwan; e Department of Pathology, National Taiwan University Hospital and National Taiwan University College of Medicine, Taipei, Taiwan

**Keywords:** Charlson Comorbidity Index, melanoma, neutrophil-to-lymphocyte ratio, prognosis, sentinel lymph node biopsy

*To the Editor:* Pretreatment neutrophil-to-lymphocyte ratio (NLR) is widely reported as an adverse prognostic marker in melanoma.^1^ However, systemic inflammation may also reflect noncancer comorbidities—known to influence cancer outcomes^2^^,^^3^ and linked to melanoma prognosis.^4^^,^^5^ This raises the question of whether elevated NLR primarily reflects comorbidity burden rather than melanoma aggressiveness. We therefore evaluated the prognostic value of pretreatment NLR while explicitly examining its relationship with the Charlson Comorbidity Index (CCI), with sentinel lymph node (SLN) status assessed in subgroup analyses.

We conducted a single-center retrospective cohort study of 288 patients with histologically confirmed melanoma at National Taiwan University Hospital (2001-2022) with available pretreatment complete blood count (Supplementary Fig 1, available via Mendeley at https://data.mendeley.com/datasets/y5pkpj3g42/1). Patients with conditions likely to distort inflammatory indices or with insufficient data were excluded. Survival outcomes were assessed using Kaplan–Meier and Cox regression. Univariable Cox analyses assessed sex, age, melanoma subtype, Breslow thickness, ulceration, lymph node metastasis, M stage, pretreatment NLR, and CCI; variables with *P* < 0.05 were entered into multivariable models. (See Supplementary Methods, available via Mendeley at https://data.mendeley.com/datasets/y5pkpj3g42/1).

The cohort was acral lentiginous melanoma–predominant (214/288, 74.3%) with a mean follow-up of 4.63 years (Supplementary Table I, available via Mendeley at https://data.mendeley.com/datasets/y5pkpj3g42/1). The optimal NLR cut-off for melanoma-specific death was 2.8 (Supplementary Fig 2, available via Mendeley at https://data.mendeley.com/datasets/y5pkpj3g42/1). Elevated NLR was not explained by comorbidity burden, as CCI distribution was similar across NLR groups (*P* = 0.210, Table I). In contrast, high NLR was associated with adverse tumor characteristics, including ulceration (*P* = 0.018), M1 disease at diagnosis (*P* = 0.016), and AJCC stage III-IV (*P* = 0.009). Consistent with this, CCI was not associated with melanoma-specific survival in univariable analysis. In multivariable Cox regression adjusting for Breslow thickness, ulceration, lymph node metastasis, and M stage (Table II), high NLR remained independently associated with melanoma-specific mortality. Similar associations were observed for disease-free survival and distant metastasis-free survival (Supplementary Tables II, III, and Supplementary Fig 3, available via Mendeley at https://data.mendeley.com/datasets/y5pkpj3g42/1), with high NLR remaining significant in multivariable model after adjustment for age, Breslow thickness, ulceration, and lymph node metastasis for disease-free survival, and for Breslow thickness, ulceration, and lymph node metastasis for distant metastasis-free survival.

**Table I:** Clinicopathologic characteristics of the 288 enrolled patients with melanoma

Characteristics		Total (*n*, %)	High NLR (≥2.8, *n* = 80)	Low NLR (<2.8, *n* = 208)	*P* value
Age (y)		63.98 ± 16.29	70.15 ± 16.62	61.61 ± 15.57	<.001∗
Gender	Female	146 (50.7%)	37	109	.36
	Male	142 (49.3%)	43	99	
Subtype	Nonacral	74 (25.7%)	18	56	.547
	Acral	214 (74.3%)	62	152	
Breslow thickness (mm)	Tis	39 (13.5%)	9	30	.178
	≤1 mm	53 (18.4%)	17	36	
	1.1-2.0 mm	57 (19.8%)	11	46	
	2.1-4.0 mm	59 (20.5%)	14	45	
	>4 mm	80 (27.8%)	29	51	
Ulceration	No	190 (66.0%)	44	146	.018†
	Yes	98 (34.0%)	36	62	
Lymph node metastasis	No	225 (78.1%)	56	169	.055
	Yes	63 (21.9%)	24	39	
M stage	M0	279 (96.9%)	74	205	.016†
	M1	9 (3.1%)	6	3	
Mitotic rate (per mm^2^)‡	<1	63 (21.9%)	13	50	.238
	1-5	101 (35.1%)	33	68	
	>5	39 (13.5%)	12	27	
Tumor-infiltrating lymphocytes‡	None	19 (6.6%)	7	12	.538
	Nonbrisk	150 (52.1%)	40	110	
	Brisk	6 (2.1%)	1	5	
Lymphovascular invasion∗	No	162 (56.2%)	44	118	.570
	Yes	17 (5.9%)	6	11	
Perineural invasion‡	No	153 (87.4%)	44	109	.444
	Yes	22 (12.6%)	4	18	
Regression‡	No	130 (45.1%)	39	91	.539
	Partial	36 (12.5%)	8	28	
	Extensive	1 (0.3%)	0	1	
Desmoplasia‡	No	153 (53.1%)	45	108	.182
	Yes	12 (4.2%)	1	11	
Disease status at presentation§	Localized disease‖	261 (90.6%)	68	193	.068
	Lymph node metastasis¶	27 (9.4%)	12	15	
AJCC stage at diagnosis	Stages 0-2	219 (76.0%)	52	167	.009‖
	Stages 3-4	69 (24.0%)	28	41	
Charlson Comorbidity Index	0	150 (52.1%)	36	114	.210
	1-2	89 (30.9%)	26	63	
	≥3	49 (17.0%)	18	31	

*NLR*, Neutrophil-to-lymphocyte ratio.

∗ *P* < 0.05.

† *P* < 0.01.

‡ Available data only.

§ Nine patients with M1-stage disease were excluded from analysis.

‖ Microscopic nodal or localized disease.

¶ Clinical or radiographic lymph node metastasis.

**Table II:** Univariable and multivariable Cox regression analyses for melanoma-specific survival (288 patients)

Variables		Univariable analysis		Multivariable analysis	
		HR (95% CI)	*P* value	HR (95% CI)	*P* value
Gender	Female	1			
	Male	1.291 (0.787-2.120)	.31		
Age (y)	<50	1			
	50-70	1.261 (0.591-2.693)	.55		
	≥70	1.818 (0.855-3.863)	.12		
Subtype	Non-Acral	1			
	Acral	1.314 (0.713-2.420)	.38		
Breslow thickness (mm)	Tis	1		1	
	≤1	4.863 (0.585-40.398)	.14	3.131 (0.374-26.235)	.293
	1-2	3.868 (0.466-32.132)	.21	2.745 (0.327-23.036)	.352
	2-4	12.244 (1.623-92.382)	.015∗	7.998 (1.034-61.852)	.046∗
	>4	24.51 (3.346-179.552)	.002†	10.553 (1.369-81.320)	.024∗
Ulceration	No	1		1	
	Yes	3.545 (2.142-5.869)	<.001†	1.530 (0.865-2.706)	.144
Lymph node metastasis	No	1		1	
	Yes	4.863 (2.943-8.035)	<.001†	2.706 (1.572-4.657)	<.001†
Distant metastasis (M stage)	M0	1		1	
	M1	14.51 (5.501-38.270)	<.001†	5.729 (2.058-15.947)	<.001†
NLR	<2.8	1		1	
	≥2.8	3.568 (2.166-5.877)	<.001†	3.064 (1.803-5.206)	<.001†
CCI	0	1			
	1-2	0.808 (0.464-1.405)	.450		
	≥3	0.468 (0.208-1.05)	.066		

Significant differences denoted by asterisks.

*CCI*, Charlson Comorbidity Index; *CI*, confidence interval; *HR*, hazard ratio; *NLR*, neutrophil-lymphocyte ratio.

∗ *P* < 0.05.

† *P* < 0.01.

Among 225 patients who underwent SLN biopsy, 54 (24.0%) had positive SLN (Supplementary Table IV, available via Mendeley at https://data.mendeley.com/datasets/y5pkpj3g42/1). In 171 SLN-negative patients, where comorbidity-related confounding may be most clinically relevant, CCI again showed no association with melanoma-specific survival. In contrast, high NLR remained independently associated with melanoma-specific survival after adjustment for ulceration and Breslow thickness in multivariable model (Supplementary Table V, available via Mendeley at https://data.mendeley.com/datasets/y5pkpj3g42/1). High NLR also remained independently associated with disease-free survival (adjusted for age, Breslow thickness, and ulceration) and distant metastasis-free survival (adjusted for Breslow thickness and ulceration) in SLN-negative patients in multivariable model (Supplementary Tables VI, VII, and Supplementary Fig 4, available via Mendeley at https://data.mendeley.com/datasets/y5pkpj3g42/1).

In summary, although comorbidity burden can influence cancer outcomes, pretreatment NLR in our cohort provided prognostic information beyond standard clinicopathologic factors and was not explained by CCI. Notably, NLR further stratified risk even among SLN-negative patients, supporting its potential role as a simple adjunct for counseling and tailoring surveillance. Limitations include the retrospective single-center design and an acral-predominant case that may limit generalizability.

## Conflicts of interest

None disclosed.

## References

[1] Yang Y., Fang H., Cai Z., Xu Q. (2025). Prognostic significance of neutrophil-to-lymphocyte ratio in melanoma: a systematic review and meta-analysis. J Surg Oncol.

[2] Panigrahi G., Ambs S. (2021). How comorbidities shape cancer biology and survival. Trends Cancer.

[3] Sarfati D., Koczwara B., Jackson C. (2016). The impact of comorbidity on cancer and its treatment. CA Cancer J Clin.

[4] Chang C.K., Hsieh Y.S., Chen P.N. (2022). A cohort study: comorbidity and stage affected the prognosis of melanoma patients in Taiwan. Front Oncol.

[5] Grann A.F., Froslev T., Olesen A.B., Schmidt H., Lash T.L. (2013). The impact of comorbidity and stage on prognosis of Danish melanoma patients, 1987-2009: a registry-based cohort study. Br J Cancer.

